# Supplementary material for: Novel Pickering High Internal Phase Emulsion Stabilized by Food Waste-Hen Egg Chalaza
Source: Foods. 2021 Mar 12;10(3):599. doi: 10.3390/foods10030599 (PMC7998105; doi:10.3390/foods10030599)
Supplement: Supplementary file 1 [file foods-10-00599-s001.pdf]

Figure S1. Microscopicobservation of 0.3% wt chalaza suspension at different pH levels. A: 2.0; B: 3.0; C:4.0; D:5; E:6.5; F:8. The corresponding bar is 10 μm.

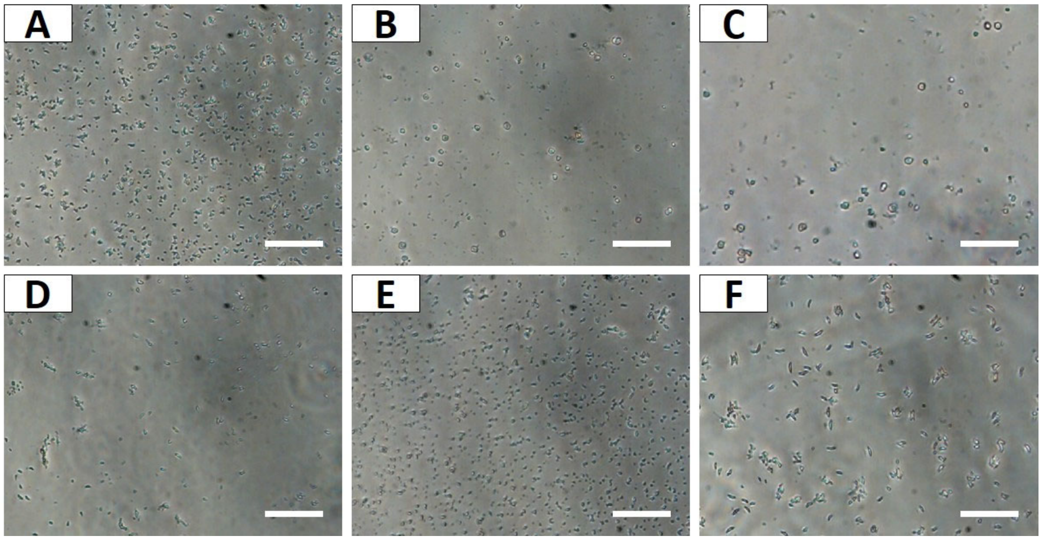

Figure S2. Microscopicobservation of 0.3% wt chalaza suspension at different ionic strength. A: 0 M; B: 0.2 M; C: 0.4 M; D: 0.6 M; E: 0.8 M; F: 1.0 M. The corresponding bar is 10 μm.

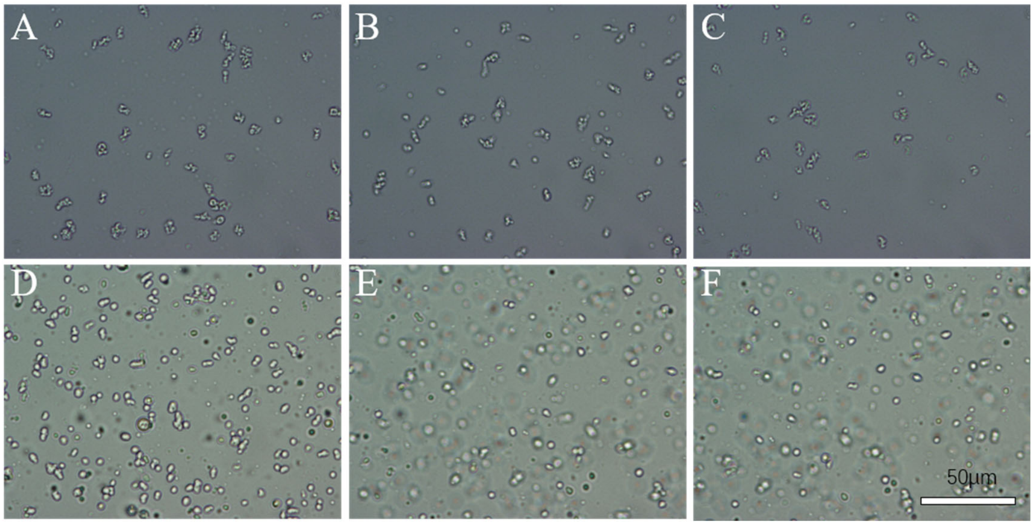

Figure S3: A: Appearance of emulsions stabilized by 1wt% chalaza at different ionic strength with 75% oil after 7days' storage;e B: Appearance of emulsions stabilized by 1wt% chalaza at different ionic strength with different oil fraction after 7 days' storage ;C: centrifugation stability of HIPEs stabilized by 1wt% chalaza at different ionic strength with 75% oil.

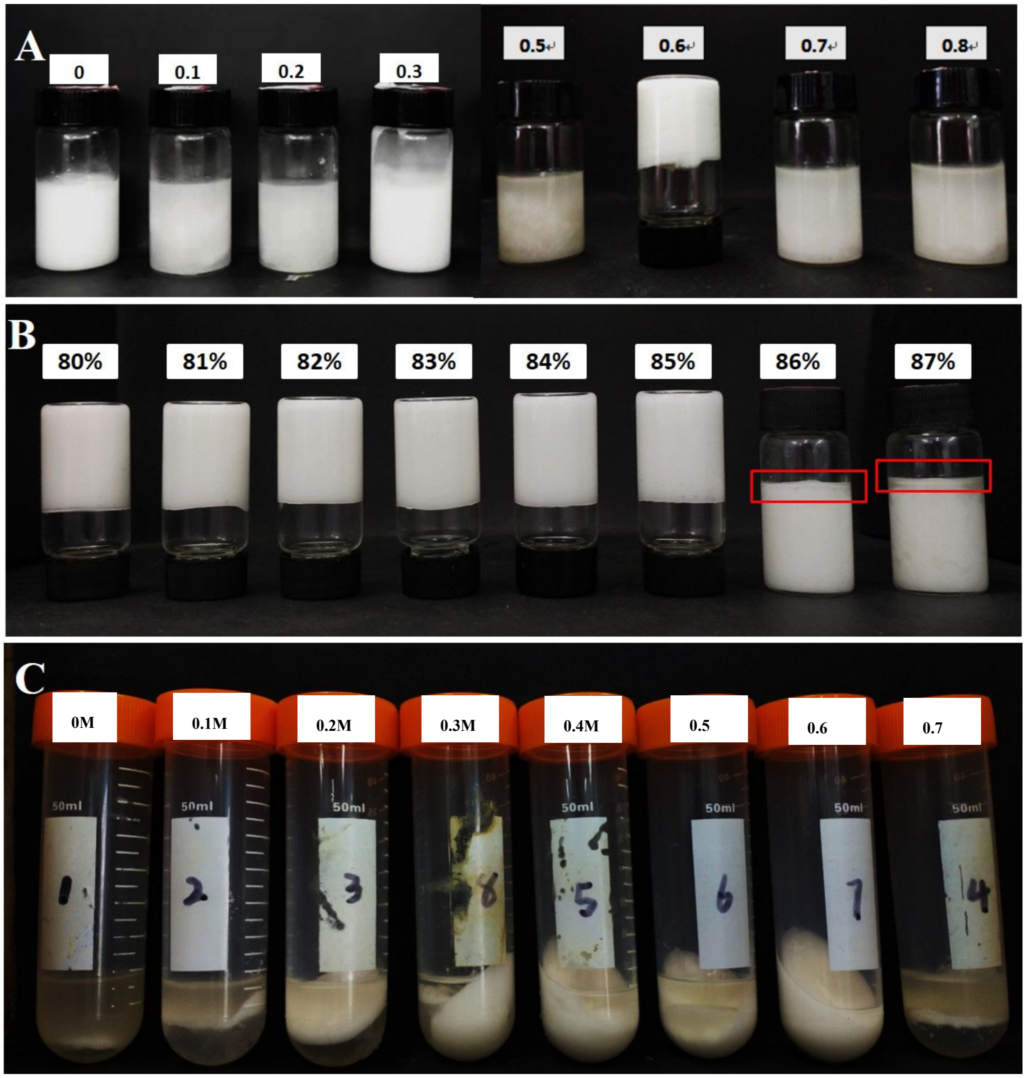
A
0
0.1
0.2
0.3
0.5
0.6
0.7
0.8
B
80%
81%
82%
83%
84%
85%
86%
87%
C
0M
0.1M
0.2M
0.3M
0.4M
0.5
0.6
0.7
